# The impact of stigma and self-esteem on quality of life after burn injury—an empirical analysis using structural equation modeling

**DOI:** 10.3389/fpsyt.2025.1616762

**Published:** 2025-10-08

**Authors:** Yanbing Liu, Xiumei Zhu

**Affiliations:** ^1^ Shaanxi Provincial People’s Hospital, Xi’an, Shaanxi, China; ^2^ Department of Burn Plastic and Cosmetic Surgery, Shaanxi Provincial People’s Hospital, Xi’an, Shaanxi, China

**Keywords:** burn patients, stigma, self-esteem, quality of life, structural equation modeling, mediating effect, stigmatization

## Abstract

**Objective:**

To explore the relationships between stigma, self-esteem, and quality of life in burn patients. This study assesses the mediating role of self-esteem between stigma and quality of life using structural equation modeling, providing a theoretical basis for improving patients’ quality of life.

**Methods:**

A convenience sampling method was used to select 264 patients in the rehabilitation phase from the burn department of Shaanxi Provincial People’s Hospital between October 2022 and October 2024. The Chinese version of the Social Impact Scale (SIS), Rosenberg Self-Esteem Scale (SES), and Burn Specific Health Scale – Brief (BSHS-B) were used to assess stigma (higher scores indicate stronger stigma), self-esteem (higher scores indicate higher levels of self-esteem), and quality of life (lower scores indicate better quality of life). Pearson correlation analysis was used to explore the correlations among the variables, and structural equation modeling was employed to analyze the mediating role of self-esteem.

**Results:**

The total scores for stigma (M = 61.21, SD = 11.58), self-esteem (M = 26.28, SD = 5.24), and quality of life (M = 61.26, SD = 10.58) were found, with significant differences across gender, work status, primary source of medical expenses, and burn severity (*P*<0.05). Pearson correlation analysis showed that stigma was significantly negatively correlated with both self-esteem and quality of life (*P*<0.01), while self-esteem was significantly positively correlated with quality of life (*P*<0.01). Structural equation modeling indicated that self-esteem played a partial mediating role between stigma and quality of life (accounting for 28.37% of the total effect), with good model fit.

**Conclusion:**

Stigma, self-esteem, and quality of life are closely related in burn patients, with self-esteem playing a significant mediating role. Reducing stigma and enhancing self-esteem are important strategies for improving patients’ quality of life, providing a basis for psychological interventions.

## Introduction

Burn injury is a traumatic condition with profound impacts on patients’ physical health and psychological well-being. It not only causes severe functional impairment but may also result in permanent cosmetic alterations, significantly affecting patients’ daily activities and social interactions (1, 2). Such external damage is often accompanied by psychological issues such as inferiority, anxiety, and depression, which can further diminish treatment compliance and quality of life (3). In recent years, advances in medical technology have significantly improved burn treatment in areas such as emergency care, skin grafting, and scar repair, enabling more patients to survive and progress to the rehabilitation phase (4–6). However, the rehabilitation phase demands not only prolonged physical recovery but also confronting psychological pressures stemming from altered appearance and social adaptation. While existing research predominantly focuses on the direct physical and cosmetic impacts of burns, studies on the mental health of burn patients—particularly the role of stigma and self-esteem in quality of life—remain insufficient. Research indicates that psychological health issues are critical factors affecting burn patients’ rehabilitation quality and life satisfaction, with stigmatization and self-esteem playing pivotal roles in patients’ psychological adaptation and social functional recovery (7, 8). Therefore, exploring key influencing mechanisms in burn patient rehabilitation from a mental health perspective holds significant importance for comprehensively enhancing their quality of life.

Stigma refers to the emotional and cognitive state where individuals experience shame, social discrimination, or self-deprecation due to a disease or physical impairment. This psychological phenomenon typically arises from the interplay of disease characteristics, sociocultural perceptions, and patients’ subjective experiences. Its core manifestations include negative perceptions of disease-related conditions, resulting in social interaction barriers and psychological adaptation difficulties (9, 10). Stigma significantly negatively impacts patients’ mental health, social functioning, and quality of life. Research indicates that individuals with cancer (11), depression (12), and HIV/AIDS (13) often experience pronounced stigmatization due to disease characteristics and societal prejudice. They may face external discrimination and bias while simultaneously experiencing self-deprecation stemming from the disease’s unique nature, leading to heightened social isolation and feelings of uncertainty and helplessness about the future. Stigma significantly diminishes patients’ psychological coping abilities, impairs treatment adherence, and further exacerbates declines in quality of life (14). The study by Wu et al. (15) found a moderate positive correlation between stigma and low self-esteem in burn patients (*r* = 0.546, *P*< 0.001). Factors such as place of residence, itching, and self-esteem scale scores were significant influences on stigma, collectively explaining 38.5% of the total variance in stigma. Although stigma has been extensively studied across various diseases, research exploring the relationship between stigma and quality of life in burn patients remains insufficient. Specifically, how stigma affects psychological adaptation and social functional recovery in burn patients is a critical topic for improving their mental health and living conditions.

In contrast to stigma, self-esteem represents a positive psychological resource. It reflects an individual’s affirmation or negation of their inherent worth and capabilities, encompassing cognitive and emotional experiences related to one’s body, abilities, personality traits, and social roles (16). Low self-esteem often manifests as psychological issues such as depression, anxiety, and social phobia; conversely, high self-esteem is typically associated with greater psychological resilience, reduced incidence of depression and anxiety, and higher life satisfaction. Research by Vrbová et al. (17) indicates that individuals with schizophrenia and comorbid social phobia exhibit lower self-esteem and poorer quality of life. For burn patients, self-esteem is often negatively impacted by multiple factors including physical disfigurement, functional limitations, and social discrimination. Scarring and deformities may damage self-image, triggering doubts about personal worth. Concurrently, societal biases and stereotypes regarding physical changes further exacerbate psychological distress, leading to diminished self-esteem (18). The study by Gorbani et al. (19) found that low self-esteem not only significantly reduced the rehabilitation motivation and treatment adherence of burn patients but also had long-term negative effects on their emotional, interpersonal, and occupational functioning. A follow-up study by Deng et al. (20) showed that enhancing positive psychological resources could alleviate social avoidance and improve life satisfaction in burn patients. However, this study did not include stigma as a core analysis variable and therefore did not further explore its potential role. Although self-esteem is considered a key factor in improving quality of life in various diseases, there is still insufficient research on how self-esteem affects the quality of life in burn patients. However, investigating the level of self-esteem in burn patients is of significant practical importance.

In this study, we chose self-esteem as a mediator rather than a moderator or predictor variable. The core rationale is based on social cognitive theory and self-determination theory. The former suggests that an individual’s cognitive processing of external evaluations, such as stigma, influences their self-concept (with self-esteem as a core dimension), which in turn shapes psychological experiences related to quality of life. The latter indicates that stigma undermines an individual’s sense of belonging and autonomy, leading to a decrease in self-esteem and weakening the perception of life’s meaning, ultimately reducing quality of life. As a mediator, self-esteem can reveal the path through which stigma influences quality of life via internal psychological resource depletion, whereas a moderator only explains changes in the strength of the relationship without clarifying the direct mechanism. Therefore, self-esteem is not suitable as a moderator.

Current research primarily focuses on the impact of burns on physical function and appearance, with insufficient exploration of the mechanisms linking stigma, self-esteem, and quality of life. Based on this, the present study proposes the following hypotheses: (1) Stigma is negatively correlated with both self-esteem and quality of life; (2) Self-esteem is positively correlated with quality of life; (3) Self-esteem mediates the relationship between stigma and quality of life. The study aims to construct a structural equation model (SEM) to analyze the relationships between these three variables and the mediating role of self-esteem, filling the gap in related research and providing theoretical support for improving the quality of life in burn patients.

## Materials and methods

### Study population

This study employed convenience sampling, selecting burn patients undergoing inpatient rehabilitation and outpatient follow-up at the Burn Department of Shaanxi Provincial People’s Hospital between October 2022 and October 2024. Inclusion criteria: age 18–60 years; in the burn rehabilitation phase with mild, moderate, or severe burn severity; conscious and able to complete questionnaires; voluntary participation with signed informed consent. Exclusion criteria: severe chronic comorbidities; severe psychiatric disorders or cognitive impairment; physical disabilities unrelated to burns; refusal to participate or non-cooperation. This study was approved by the Medical Ethics Committee of Shaanxi Provincial People’s Hospital.

### Sample size calculation

This study determined the sample size based on the requirements of structural equation modeling (SEM) analysis, ensuring its rationality through a combination of “empirical estimation and professional tool verification.” Basic Range Estimation: The research model includes three latent variables—stigma, self-esteem, and quality of life—corresponding to 24 core observed variables after item packaging. According to the classical empirical rule for SEM sample size estimation (observed variable count × 5 to 20), 24 observed variables require a sample size of 120 to 480 cases. Additionally, the three latent variables require at least 150 cases. Based on this, the preliminary sample size range was determined to be between 150 and 480 cases. Professional Tool Effectiveness Analysis: To further verify this estimation, statistical power analysis was conducted using G*Power 3.1.9.7 and the pwrSEM package (R language). Parameter settings referenced Wu et al. (2023) (15), who studied the relationship between stigma and self-esteem in burn patients, as well as the pre-experimental data of this study (the total effect of stigma on quality of life, β=-0.21). The expected effect size for the mediating effect was set at f²=0.12 (medium effect size), with a power of (1-β)=0.90 and a significance level α=0.05 (two-tailed). The analysis showed that the minimum sample size required for model parameter estimation was 212 cases. When the sample size reached ≥250 cases, the model fit indices (RMSEA ≤ 0.08, CFI ≥ 0.90) met the target at a rate of ≥95%, and the coverage of the mediating effect confidence interval was ≥92%, ensuring the reliability of the results. Final Sample Size Determination: Considering the potential for sample attrition (such as incomplete questionnaires) during the study, an attrition rate of 10% was anticipated. Therefore, the final sample size was set at 264 patients. This sample size falls within the empirically estimated range and has been validated through professional tools, meeting the requirements for model fitting and mediating effect testing.

### Survey tools

#### Stigma

The Chinese version of the Social Impact Scale (SIS) (21) was used to assess patients’ stigmatization. This 24-item scale measures four dimensions: social exclusion, economic deprivation, internalized shame, and social isolation. Each item employs a 4-point Likert scale (1=Strongly disagree, 2=Disagree, 3=Agree, 4=Strongly agree). The total score ranges from 24 to 96 points, with higher scores indicating greater levels of stigmatization. Low stigmatization: 24–48 points; moderate stigmatization: 49–72 points; high stigmatization: 73–96 points. The Cronbach’s α coefficient for this scale is 0.875.

#### Self-esteem

The Chinese version of the Rosenberg Self-Esteem Scale (SES) (22) was used to assess burn patients’ self-esteem. This scale consists of 10 items, each rated on a 4-point Likert scale (1=Strongly disagree, 2=Disagree, 3=Agree, 4=Strongly agree). The total score ranges from 10 to 40 points, with higher scores indicating greater self-esteem. Low self-esteem: 10–25 points; Moderate self-esteem: 26–32 points; High self-esteem: 33–40 points. The Cronbach’s α coefficient for this scale is 0.884.

#### Quality of life

Quality of life among burn patients was assessed using the Chinese version of the Burn Health Scale-Brief (BSHS-B) (23). This scale comprises 40 items across nine dimensions: basic activities of daily living, hand function, emotional well-being, interpersonal relationships, sexual life, body image, thermal sensitivity, treatment compliance, and work capacity. Each item is rated using a 5-point Likert scale (1=Strongly Disagree, 2=Very Disagree, 3=Somewhat Disagree, 4=Somewhat Agree, 5=Strongly Agree). In this scale, lower scores indicate better quality of life, while higher scores indicate poorer quality of life. Quality of Life Grading: High Quality of Life: Total score ≥ 70% (i.e., total score ≥ 140 points). Moderate quality of life: Total score percentage between 50%–69% (i.e., 100 points ≤ total score<140 points). Low quality of life: Total score percentage<50% (i.e., total score<100 points). The Cronbach’s α coefficient for this scale is 0.860.

### Statistical methods

Data analysis was conducted using SPSS 27.0 and AMOS 24.0. Descriptive statistics were applied to examine participants’ sociodemographic characteristics, stigma of illness (SIS score), self-esteem (SES score), and quality of life (BSHS-B score). Continuous variables are presented as mean ± standard deviation (Mean ± SD), while categorical variables are reported as frequency and percentage [*n* (%)]. For intergroup comparisons, continuous variables were analyzed using t-tests or one-way analysis of variance (ANOVA), while categorical variables were assessed using chi-square tests. Pearson correlation analysis explored relationships among disease stigma, self-esteem, and quality of life indicators. Statistical significance was set at P<0.05. In structural equation modeling (SEM) analysis, we employed maximum likelihood estimation (MLE), which assumes data normality. When normality was violated, robust maximum likelihood estimation (MLR) was used to handle non-normally distributed data and provide robust estimates. Model fit was assessed using the chi-square value divided by degrees of freedom (χ²/df), root mean square error of approximation (RMSEA), comparative fit index (CFI), and inflection-based fit index (IFI). Acceptable fit criteria were: 2/df ≤ 3, RMSEA ≤ 0.08, CFI ≥ 0.90, and IFI ≥ 0.90. Indirect effects were calculated using bootstrapping with 5000 resamples, reporting bootstrap standard errors (BootSE) and 95% confidence intervals (Boot95%CI). Mediating effect significance was determined by whether the confidence interval included zero. All statistical analyses were set at a *two-tailed* significance level of P<0.05.

## Results

### Baseline characteristics of burn patients

Baseline data revealed a predominantly male patient population, with ages predominantly concentrated between 18 and 44 years, indicating a relatively young demographic. Most patients were employed, though a portion were unemployed or retired. Primary sources of medical expense coverage were urban and rural resident medical insurance, with some patients relying on urban employee medical insurance, work injury insurance, or other channels. Regarding burn causes, thermal burns were most common, followed by electrical and chemical burns, with other causes being relatively rare. Multiple-site burns were prevalent, with upper limbs, hands, and head/neck/face being high-incidence areas. Most burns were mild to moderate in severity, with severe burns accounting for a smaller proportion (**Table 1**).

**Table 1:** Baseline data of burn patients.

Variable	Category	Frequency (n)	Percentage (%)
Gender	Male	182	68.94
	Female	82	31.06
Age (years)	18~44	166	62.88
	45~60	98	37.12
Education Level	Junior High School or below	180	68.18
	Vocational/High School	47	17.80
	College or above	37	14.02
Marital Status	Married	180	68.18
	Single	84	31.82
Employment Status	Employed	164	62.12
	Unemployed/Retired	100	37.88
Main Source of Medical Expenses	Urban and Rural Residents’ Insurance	175	66.29
	Urban Employee Insurance	55	20.83
	Work Injury Insurance	21	7.96
	Others	13	4.92
Burn Cause	Thermal	190	71.97
	Chemical	24	9.09
	Electrical	42	15.91
	Other	8	3.03
Burn Area	Head/Neck/Face	121	45.83
	Hands	132	50.00
	Upper Limbs	137	51.89
	Lower Limbs	74	28.03
	Trunk	85	32.20
	Multiple Areas	150	56.82
Burn Severity	Mild	127	48.11
	Moderate	100	37.88
	Severe	37	14.01

### Descriptive analysis of stigma, self-esteem, and quality of life

The mean score for stigmatization of illness (SIS scale) among burn patients was 61.21 ± 11.58, with 53, 172, and 39 patients exhibiting low, moderate, and high self-esteem, respectively. The mean self-esteem score (SES scale) was 26.28 ± 5.24, with 169, 38, and 57 patients classified as low, moderate, and high self-esteem, respectively. The mean score for quality of life (BSHS-B scale) was 61.26 ± 10.58, with 36, 192, and 36 patients reporting low, moderate, and high quality of life, respectively (**Table 2**).

**Table 2:** Descriptive scores of stigma, self-esteem, and quality of life in burn patients.

Variable	Mean score
SIS Scale	61.21 ± 11.58
SES Scale	26.28 ± 5.24
BSHS-B Scale	61.26 ± 10.58

**Figure 1:**
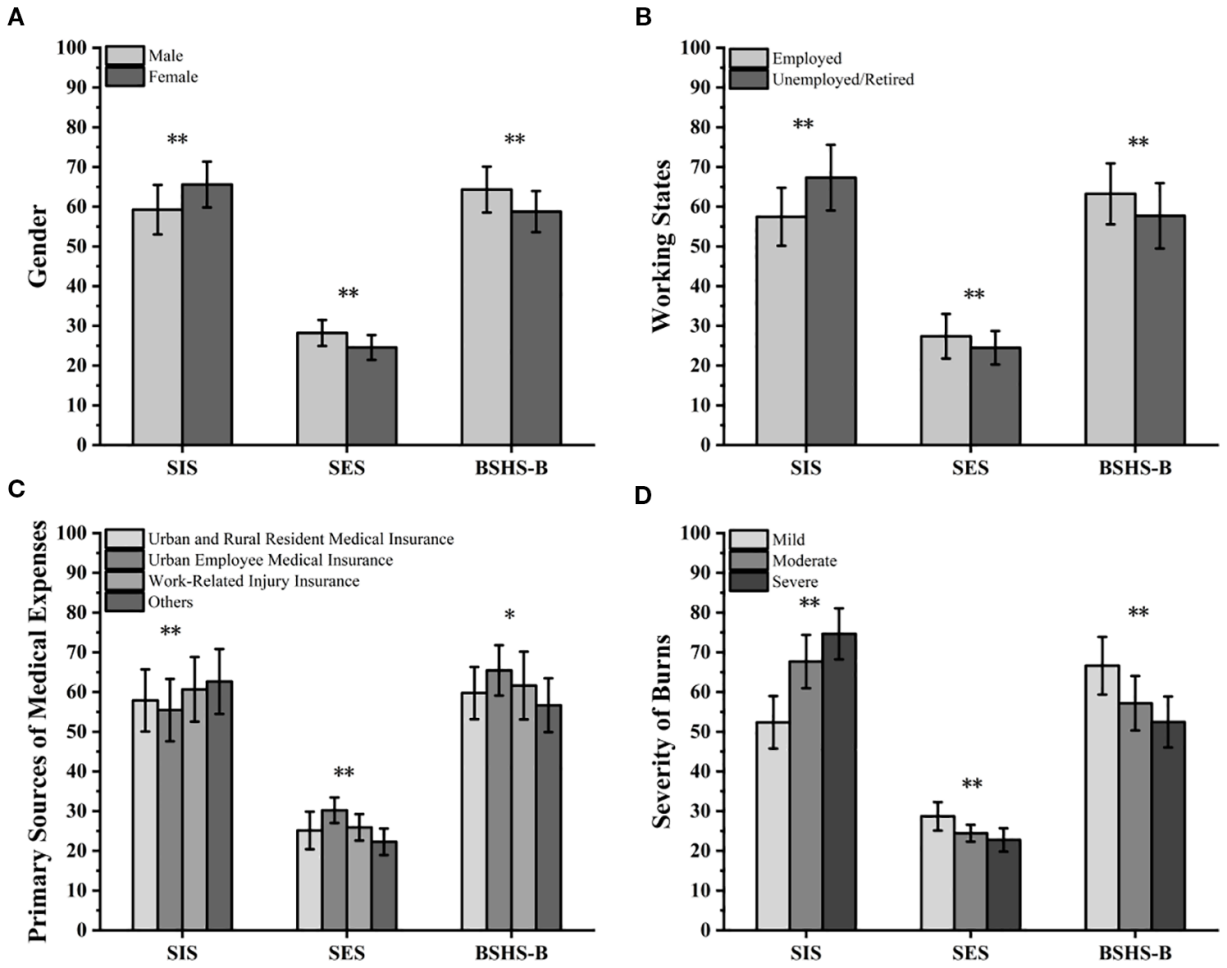
Differences in SIS, SES, and BSHS-B under different factors: **(A)** Different genders; **(B)** Different work status; **(C)** Different sources of medical expense coverage; **(D)** Different burn severity.**P<0.01, *P<0.05.

**Figure 1** shows that various factors significantly influence SIS, SES, and BSHS-B scores in burn patients. Male burn patients had significantly higher SIS scores than females (t=3.217; P = 0.001), while females had significantly higher SES (t=2.894; P = 0.004) and BSHS-B (t=2.658; P = 0.008) scores than males (all P<0.05). Employed patients exhibited lower SIS scores (t=2.689; *P* = 0.008), while SES (t=2.635; *P* = 0.009) and BSHS-B (t=2.753; *P* = 0.006) scores were significantly higher than those of unemployed or retired patients. Patients whose medical expenses were primarily covered by urban and rural resident medical insurance had higher SIS scores and lower SES and BSHS-B scores, whereas those covered by urban employee medical insurance and work injury insurance exhibited better psychological status and quality of life. Overall differences between groups with different medical expense sources were statistically significant (SIS: F = 4.215, *P* = 0.006; SES: F = 4.158, *P* = 0.006; BSHS-B: F = 2.*945, P* = 0.034). The more severe the burn injury, the higher the SIS (F = 7.538; P = 0.001), while SES (F = 6.583; *P* = 0.002) and BSHS-B (F = 7.512; *P* = 0.001) were significantly lower.

### Correlation between dimensions of burn patients’ stigma, self-esteem, and quality of life

Research findings indicate a significant correlation among stigmatization, self-esteem, and quality of life among burn patients. Stigmatization exhibited a significant negative correlation with self-esteem (*P<*0.01), with the social isolation dimension of stigmatization exerting the most pronounced negative impact on self-esteem (r=-0.354). Self-esteem showed a significant positive correlation with quality of life (*P<*0.01), with particularly pronounced positive effects on basic activities of daily living, hand function, work capacity, and body image. Stigma was significantly negatively correlated with quality of life (*P<*0.01), with the social isolation dimension of stigma exerting the most pronounced negative impact (r=-0.441), particularly affecting sexual life, body image, and interpersonal relationships (**Table 3**).

**Table 3:** Pearson correlation analysis between stigmatization, self-esteem, and various dimensions of quality of life in burn patients.

Variable	SIS total score	Social exclusion	Economic discrimination	Intrinsic shame	Social isolation	SES total score	BSHS-B total score	Basic life skills	Hand function	Emotional	Interpersonal relationships	Sexual function	Body image	Heat sensitivity	Cooperation with treatment	Work
SIS Total Score	1	-0.413**	-0.322**	-0.268**	-0.354**	-0.413**	-0.423**	-0.258**	-0.266**	-0.240**	-0.331**	-0.308**	-0.314**	-0.278**	0.045	-0.132*
Social Exclusion		1	0.681**	0.545**	0.602**	0.322**	0.348**	0.228**	0.271**	0.181**	0.253**	0.205**	0.277**	0.238**	-0.052	0.067
Economic Discrimination			1	0.753**	0.510**	0.268**	0.176**	0.113	0.087	0.074	0.256**	0.095	0.131**	-0.037	0.145*	0.058
Intrinsic shame				1	0.621**	0.312**	0.250**	0.161*	0.025	0.178**	0.231**	0.282**	0.210**	0.192**	-0.021	0.071
Social isolation					1	0.354**	0.441**	0.261**	0.312**	0.257**	0.273**	0.328**	0.283**	-0.332**	-0.097	0.211**
SES Total Score						1	0.517**	0.358**	0.371**	0.173**	0.301**	0.297**	0.318**	0.282**	0.136**	0.334**
BSHS-B Total Score							1	0.517**	0.371**	0.301**	0.334**	0.318**	0.283**	0.332**	0.136**	0.334**
Basic Living Skills								1	0.358**	0.301**	0.334**	0.282**	0.318**	0.282**	0.136**	0.334**
Hand Function									1	0.173**	0.301**	0.318**	0.318**	0.282**	0.136**	0.334**
Emotion										1	0.231**	0.282**	0.257**	0.238**	0.136**	0.334**
Interpersonal Relationships											1	0.328**	0.283**	0.332**	0.136**	0.334**
Sexual Activity												1	0.283**	0.332**	0.136**	0.334**
Body image													1	0.332**	0.136**	0.334**
Thermosensitive														1	0.136**	0.334**
CombinationTherapy															1	0.334**
Work																1

**P<0.01, *P<0.05.

### The mediating effect of self-esteem on the relationship between stigma and quality of life in burn patients

**Figure 2:**
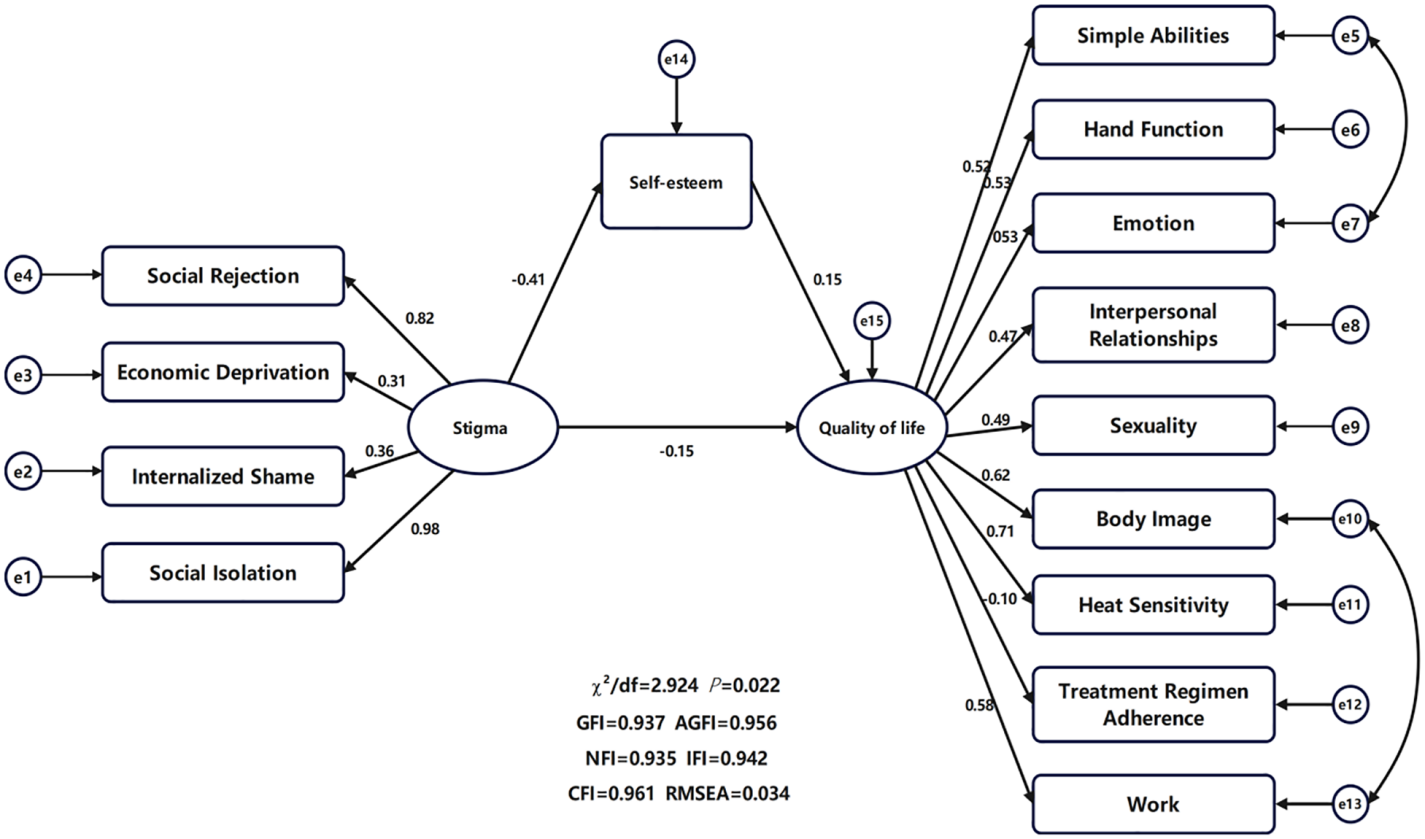
Mediation model of self-esteem between stigma and quality of life.

This study constructed a structural equation model with stigmatization as the independent variable, self-esteem as the mediating variable, and quality of life as the dependent variable. Model fit indices indicated that the model had good fit:χ^2^/df<3, with GFI, AGFI, NFI, IFI, and CFI all ≥ 0.9, and RMSEAS ≤ 0.08 (**Table 4**). Mediation analysis revealed that self-esteem significantly mediated the relationship between stigmatization and quality of life among burn patients. The total effect of stigmatization on quality of life was -0.214, comprising a direct effect of -0.149 (71.63% of the total effect) while the indirect effect was -0.063, accounting for 28.37%. The Bootstrap method confirmed statistical significance with *P*<0.001. The Bootstrap confidence interval (-0.115, -0.236) excluded zero, indicating the indirect effect’s statistical significance. Furthermore, stigmatization significantly reduced patients’ self-esteem (effect size=-0.410, *P<*0.001), while self-esteem significantly improved quality of life (effect size=0.154, *P<*0.001). This indicates that self-esteem partially mediates the relationship between stigmatization and quality of life. Reducing stigmatization and enhancing self-esteem are important pathways to improving the quality of life for burn patients (**Table 5**; **Figure 2**).

**Table 4:** Model fit indices of the structural equation model.

Fit index	χ^2^/df	GFI	AGFI	NFI	IFI	CFI	RMSEA	*P*
Evaluation Criteria	≤3	≥0.9	≥0.9	≥0.9	≥0.9	≥0.9	≤0.08	>0.05
Model Results	2.926	0.937	0.956	0.935	0.942	0.961	0.034	0.022

Df, Degrees of freedom; GFI, Goodness of Fit Index; AGFI, Adjusted Goodness of Fit Index; NFI, Normed Fit Index, IFI, Incremental Fit Index; CFI, Comparative Fit Index; RMSEA, Root Mean Square Error of Approximation.

**Table 5:** Mediation effect of self-esteem between stigma and quality of life in burn patients.

Model path	Mediation effect value	Boot SE	Boot 95% CI	P-value	Effect proportion
Total Effect				<0.001	100%
Stigma → Quality of Life	-0.214	0.042	(-0.287,-0.132)		
Direct effect				<0.001	71.63%
Stigma → Self-esteem	-0.410	0.073	(-0.187,-0.092)		
Stigma → Quality of Life	-0.149	0.035	(-0.356,-0.216)		
Self-esteem → Quality of life	0.154	0.032	(0.246,0.543)		
Indirect effect				<0.001	28.37%
Self-esteem → Quality of Life	-0.063	0.021	(-0.115,-0.236)		

## Discussion

Burns are a severe traumatic condition that affects not only the patient’s physical function but also their psychological health and social adaptation. Despite significant advances in modern medical technology that have greatly improved the survival rate of burn patients, the rehabilitation phase remains fraught with challenges. During recovery, patients must not only restore their physical functions but also cope with the psychological stress and social interaction difficulties caused by changes in appearance. These challenges often significantly reduce their quality of life and further hinder the reconstruction of social function (24). This study focuses on burn patients and, through analyzing the mediating effect of self-esteem between stigma and quality of life, provides new perspectives and foundations for optimizing psychological interventions and rehabilitation strategies for burn patients.

### Overall levels and influencing factors of stigma, self-esteem, and quality of life in burn patients

In this study, the overall scores of stigma (M = 61.21, SD = 11.58), self-esteem (M = 26.28, SD = 5.24), and quality of life (M = 61.26, SD = 10.58) suggest that burn patients generally experience moderate stigma and quality of life levels, with relatively low self-esteem. Additionally, factors such as gender, employment status, primary source of medical expenses, and burn severity significantly influenced stigma, self-esteem, and quality of life in burn patients (P<0.05).

Gender Factor Influence: Zahid et al. (25) found that gender and occupational status are important factors influencing self-esteem. Female patients had significantly lower self-esteem than male patients, which is closely related to social support and life satisfaction. Due to societal expectations regarding appearance and family roles, female patients are more prone to shame and inferiority when faced with appearance changes and functional limitations. This is further exacerbated by a reduction in social support and life satisfaction, contributing to a decline in self-esteem (26). Willemse et al. (27) further elaborated on the impact of gender and burn severity on the psychological state of patients. They found that burn severity, age, and gender were significantly correlated with body image dissatisfaction. This dissatisfaction, stemming from appearance changes, further lowered self-esteem. Stigma and the fear of negative social evaluation played a crucial role in this process—particularly for female patients, who are more vulnerable to psychological fragility due to higher societal expectations regarding their appearance. This finding resonates with Zahid et al.’s (25) conclusion that female patients have lower self-esteem, further emphasizing the dual impact of “body image cognition” and “fear of social evaluation” on self-esteem.

Burn Location and Economic Factors’ Influence: Wu et al. (28) focused on facial burn patients and found that they also experienced moderate levels of stigma and lower self-esteem, which were significantly correlated. Further analysis revealed that family income, education level, and the primary source of medical expenses were key factors affecting stigma and self-esteem in facial burn patients. Patients with facial burns, due to changes in appearance, were more likely to perceive social prejudice and self-stigma. Patients with better economic conditions, higher education levels, or sufficient medical insurance had more resources to cope with appearance changes and social pressures, leading to lower levels of stigma and higher self-esteem. This finding aligns with the conclusion of this study that “the primary source of medical expenses has a significant impact on the psychological state of patients”.

Occupation Status Influence: Occupation status influences self-esteem through social role value and social support. Stable employment allows patients to enhance their self-esteem through a sense of achievement, economic independence, and social interactions, whereas unemployment or career interruptions lead to decreased self-esteem due to financial pressure and loss of social roles. Farzan et al. (29) found that burn patients with employment had significantly better quality of life than unemployed patients, as employment provides economic support and social interactions. Employment helps reduce the financial strain caused by burns, improving access to medical resources and rehabilitation. Social interactions in the workplace also help patients build a sense of belonging, reduce feelings of isolation, and enhance psychological resilience, thus improving life satisfaction.

Medical Expenses and Insurance Influence: Elalem et al. (30) showed that medical insurance significantly improved the quality of life for burn patients, especially in the rehabilitation and psychological support phases post-burn. Medical insurance reduces the financial burden on patients, enabling them to access more comprehensive medical resources and rehabilitation services, thus improving physical function and health. Insurance coverage can also reduce the anxiety and psychological stress caused by high treatment costs and increase patients’ sense of security (31). Furthermore, the psychological support services covered by insurance help patients cope with the psychological trauma and stigma following burns, promoting social adaptation. Through both economic support and psychological security, medical insurance effectively enhances burn patients’ physical and mental health, significantly improving their quality of life.

Burn Severity and Social Support Influence: Mehrabi et al.’s (32) systematic review integrated multiple factors affecting self-esteem in burn patients, showing that male patients generally have higher self-esteem than females, patients with facial burns have significantly lower self-esteem than those with burns in other body areas, and burn severity is negatively correlated with self-esteem. Importantly, the study identified “social support” as a key positive factor in improving low self-esteem and quality of life. Adequate social support helps patients alleviate the psychological stress caused by appearance changes and enhances their confidence in coping with the disease. This supports the findings of Zahid et al. (26), which emphasize “social support influencing self-esteem,” and Farzan et al.’s (28) claim that “social interactions improve quality of life,” further reinforcing the crucial role of social support in the psychological rehabilitation of burn patients. Rehan et al. (33) also found that burn severity significantly increased the risk of stigma and decreased self-esteem. Severe burns often result in noticeable scarring, deformities, and functional impairments, which can lead to negative evaluations of appearance and social anxiety, thus increasing stigma. The loss of physical function also makes patients more dependent on others in daily life, leading to feelings of helplessness and a diminished sense of worth, which further decreases self-esteem (34). Burn severity can also limit patients’ participation in social roles and return to work, leading to increased social isolation and economic pressure, further affecting their psychological state and self-esteem.

In summary, the stigma, self-esteem, and quality of life of burn patients are significantly influenced by multiple factors, including gender, employment status, economic security, and burn severity. These factors act through mechanisms such as social support, economic pressure, and psychological adaptation, significantly impacting the physical and mental health of patients.

### Correlation and mediating effect of stigma, self-esteem, and quality of life

Pearson correlation analysis showed that stigma was significantly negatively correlated with both self-esteem and quality of life (*P*<0.01), and self-esteem was significantly positively correlated with quality of life (*P*<0.01). Structural equation modeling results indicated that self-esteem played a partial mediating role between stigma and quality of life, accounting for 28.37% of the total effect, and the model fit indices were good.

Mechanism of Stigma’s Impact on Life Quality and Self-Esteem: Kim et al. (35) found that in patients with chronic mental illnesses, stigma was significantly negatively correlated with subjective life quality. The study emphasized that stigma was significantly related to psychological health and social function, which might be partially influenced by self-esteem levels. Kadam et al. (36) pointed out that low self-esteem was significantly associated with poorer quality of life and had a significant impact on patients’ mental health, increasing the risk of suicide. Maslakpak et al. (37) explored the formation mechanism and influencing dimensions of self-stigma in burn patients, identifying that negative psychological states stemmed from society’s misunderstandings and prejudices against burn patients. Patients often internalize external negative perceptions as self-denial, forming self-stigma. This self-stigma not only triggers negative emotions (such as inferiority and anxiety) but also leads to social withdrawal and strained family relationships, significantly damaging patients’ psychological health. This theoretical support aligns with the “negative impact of stigma on self-esteem” mechanism in this study and clarifies the path of “social prejudice internalized as self-denial.” Stigma may also cause social isolation, anxiety, and depression, further limiting patients’ social interactions and support systems, which in turn harms their quality of life. Moreover, stigma is significantly related to health behaviors and rehabilitation participation, which can impact overall life quality (38).

Impact and Mediating Role of Self-Esteem on Life Quality: Self-esteem is significantly related to the quality of life of burn patients. Higher self-esteem helps patients enhance psychological resilience, improve their ability to cope with disease and associated stress, and increase their sense of control and happiness in life. In contrast, lower self-esteem leads to more negative emotions, feelings of helplessness, and loneliness, further deteriorating life quality. Notably, stigma and quality of life exert reciprocal influences on each other—stigma leads to a decline in quality of life, and the worsening of quality of life, in turn, intensifies stigma, creating a vicious cycle. Huang et al. (29) found that self-esteem was a mediator between stigma and quality of life, suggesting that interventions should focus on enhancing self-esteem and reducing stigma. Mechanistically, stigma and self-esteem are significantly negatively correlated, which can further influence life quality. Specifically, stigma reduces patients’ sense of self-worth and value, triggering self-denial and self-stigmatization, weakening psychological resilience and coping abilities. The decline in self-esteem further exacerbates patients’ psychological distress, making it difficult to adapt positively to the changes brought by the disease. This low self-esteem state also reduces the sense of control and satisfaction with life, increasing negative emotional experiences and thus lowering quality of life (39).

Furthermore, self-esteem serves as a psychological buffer, modulating the negative impact of stigma on life quality. Higher self-esteem can enhance psychological resilience and recovery ability, mitigating the negative effects of stigma on quality of life (40). Thus, self-esteem plays a critical bridging role in this relationship, both as a mediating pathway through which stigma impacts life quality and as a key entry point for improving life quality.

### Intervention recommendations based on research findings

Based on the research conclusions, the study proposes the following intervention suggestions: Provide personalized psychological counseling and group therapy for burn patients, along with public education to help patients properly view changes in appearance and alleviate stigma and self-stigmatization. Develop comprehensive intervention strategies, including positive reinforcement, skills training, and vocational support, while strengthening family and community support networks to help patients rebuild confidence and improve their psychological state. Expand insurance coverage and improve financial aid to reduce the burden of treatment costs, while optimizing medical services to ensure patients receive continuous physical treatment and psychological rehabilitation resources. Encourage patients to participate in public welfare activities, rehabilitation groups, and employment programs, offering social adaptation training to reduce isolation and enhance their sense of social belonging and life satisfaction.

### Study limitations and future prospects

This study has four main limitations: Single measurement method, relying only on self-report scales such as SIS, SES, and BSHS-B, which may be subject to social desirability bias. Additionally, the cross-sectional static measurements cannot capture dynamic changes in psychological states across different rehabilitation stages, potentially undermining data authenticity. Incomplete variable system, excluding key factors such as social support and emotional regulation strategies, and not exploring reverse causal relationships, which makes it difficult to comprehensively explain the mechanisms of the three variables. Limited sample representativeness, as the study only included patients from Shaanxi Provincial People’s Hospital, which may lead to regional and demographic bias. Furthermore, patients with severe mental disorders were excluded, limiting the generalizability of the results. Weak causal inference ability, as the cross-sectional design cannot clarify the temporal logic of the variables and cannot exclude false associations. Future research could improve in four areas: Optimize measurement methods by combining self-report, behavioral observation, and semi-structured interviews, and conduct longitudinal tracking for 12–24 months to dynamically capture variable changes. Refine the variable model by incorporating social support and emotional regulation, verifying causal pathways through moderation and cross-lagged models. Expand the sample size through multi-center sampling to cover different regions and populations, including patients with mental disorders, to enhance external validity. Deepen intervention research by designing targeted programs based on the mediating effect of self-esteem and verifying their effectiveness through randomized controlled trials, providing practical evidence for clinical interventions.

## Conclusion

This study found that self-esteem partially mediated the relationship between stigma and quality of life, accounting for 28.37% of the total effect. Gender, employment status, economic security, and burn severity significantly influence patients’ psychological state and quality of life, highlighting the necessity of psychological interventions and economic support. These findings suggest that interventions aimed at reducing stigma and enhancing self-esteem may be promising strategies for improving the quality of life in burn patients. However, due to sample limitations, unclear causal relationships, and insufficient control of confounding factors, future longitudinal studies are needed to further validate these relationships. In summary, comprehensive interventions targeting psychological health, social adaptation, and economic support are recommended during the rehabilitation process.

## Data Availability

The original contributions presented in the study are included in the article/supplementary material. Further inquiries can be directed to the corresponding author.
